# The Role of Hepatitis B Core-Related Antigen

**DOI:** 10.3390/genes10050357

**Published:** 2019-05-09

**Authors:** Takako Inoue, Yasuhito Tanaka

**Affiliations:** 1 Department of Clinical Laboratory Medicine, Nagoya City University Hospital, Nagoya 467-8602, Japan; clinoue@med.nagoya-cu.ac.jp; 2 Department of Virology and Liver Unit, Nagoya City University Graduate School of Medical Sciences, Nagoya 467-8601, Japan

**Keywords:** hepatitis B core-related antigen (HBcrAg), covalently closed circular DNA (cccDNA), hepatitis B virus (HBV), chronic hepatitis B (CHB), HBV DNA

## Abstract

Hepatitis B virus (HBV) cannot be completely eliminated from infected hepatocytes due to the existence of intrahepatic covalently closed circular DNA (cccDNA). Serological biomarkers reflect intrahepatic viral replicative activity as non-invasive alternatives to liver biopsy. Hepatitis B core-related antigen (HBcrAg) is a novel biomarker that has an important role in chronic hepatitis B (CHB), because it correlates with serum HBV DNA and intrahepatic cccDNA. In clinical cases with undetectable serum HBV DNA or loss of HBsAg, HBcrAg still can be detected and the decrease in HBcrAg levels is significantly associated with promising outcomes for CHB patients. HBcrAg can predict spontaneous or treatment-induced hepatitis B envelope antigen (HBeAg) seroconversion, persistent responses before and after cessation of nucleos(t)ide analogues, potential HBV reactivation, HBV reinfection after liver transplantation, and risk of hepatocellular carcinoma progression or recurrence. In this review, the clinical applications of HBcrAg in CHB patients based on its virological features are described. Furthermore, new potential therapeutic anti-HBV agents that affect intrahepatic cccDNA are under development, and the monitoring of HBcrAg might be useful to judge therapeutic effects. In conclusion, HBcrAg might be a suitable surrogate marker beyond other HBV markers to predict the disease progression and treatment responses of CHB patients.

## 1. Introduction

Hepatitis B is a life-threatening viral infection caused by hepatitis B virus (HBV). The infection can cause acute and chronic liver disease including cirrhosis and hepatocellular carcinoma (HCC) [1]. Chronic hepatitis B (CHB) affects approximately 260 million persons worldwide [2], with an estimated 15–40% developing cirrhosis and/or HCC [3]. Despite the implementation of an effective hepatitis B vaccine, CHB is still an important worldwide health problem with a high risk of death [4]. Although most CHB patients have promising clinical outcomes, in a significant number of patients, HBV infection finally leads to cirrhosis, liver failure, or HCC [5].

The majority of new HBV infections have occurred in highly endemic regions, such as China, Southeast Asia, and sub-Saharan Africa [6]. HBV transmission can be mother-to-infant, person-to-person through open cuts, scratches, sexual contact, nosocomial, or blood-borne (sharing of infected needles or drug preparation materials), depending on the prevalence and risk groups in the area [6].

HBV cannot easily be eliminated from the liver, because relaxed circular DNA is persistently converted to covalently closed circular DNA (cccDNA) in the cellular nucleus [7,8,9]. Therefore, the present goal for HBV control is to achieve a high degree of virological suppression, preferably with hepatitis B surface antigen (HBsAg) seroclearance, leading to biochemical remission, histological improvement, and reduction of the risk of complications [10,11]. Although liver biopsy is the most accurate technique to quantify intrahepatic cccDNA and HBV DNA, it is limited because of its invasive features. As a result, non-invasive serological biomarkers are expected to be used as surrogate markers of intrahepatic viral replicative activity.

With recent developments in molecular investigations, several biomarkers associated with the natural history of CHB and effectiveness of antiviral therapy have been identified [12,13]. Conventional serological biomarkers include serum HBV DNA levels and HBsAg titers, both of which predict the risk of cirrhosis and HCC [14,15,16,17]. However, cirrhosis and HCC can still occur in patients with undetectable HBV DNA [18] and HBsAg seroclearance [19,20]. Additionally, to predict spontaneous or treatment-induced HBeAg seroconversion, essential information including persistent responses before and after cessation of nucleos(t)ide analogues, potential HBV reactivation, and HBV reinfection after liver transplantation are required to improve patient outcomes; therefore, new and effective biomarkers are still required.

Quantitative data for representative additional HBV markers including hepatitis B core-related antigen (HBcrAg) have been put into practical use recently, at least in Japan, and HBV RNA is desired to be in clinical use soon [21,22]. In the last decade, hepatic inflammation and the quantification of HBV DNA have been used to inform treatment decisions, and these guided involvements have reduced liver-related complications and death [21].

Recently, our group reported other HBV markers—high-sensitivity HBsAg assay (HBsAg-HQ) and ultra-highly sensitive HBsAg assay employing a semi-automated immune complex transfer chemiluminescence enzyme immunoassay (ICT-CLEIA) [23,24]. Because the sensitivity of ICT-CLEIA was comparable to that of HBV DNA quantification, ICT-CLEIA can be used for HBV monitoring to prevent hepatitis caused by HBV reactivation [25].

The development of precise biomarkers can improve our understanding of the natural course of HBV infection and the optimization of antiviral therapy. Furthermore, because many CHB patients treated with antiviral therapy have undetectable HBV DNA, more precise biomarkers for risk prediction are required. However, the ideal approach for antiviral therapy against HBV remains an unresolved problem. With the development of effective anti-HBV treatments, the prevention of CHB progression and decrease of HCC risk in patients with CHB have been achieved [12].

HBcrAg, a novel biomarker, has been used to support CHB monitoring and the prediction of its outcome. In this review, we describe the clinical application of a new effective biomarker, HBcrAg, including the uses of HBcrAg, the relationship between HBcrAg and other laboratory tests, its capability for the prediction of clinical outcome, especially HCC occurrence, and its role as a treatment predictor. Additionally, we provide information of promising drugs that might be effective for the elimination of HBV from cccDNA.

## 2. HBV Life Cycle

**Figure 1:**
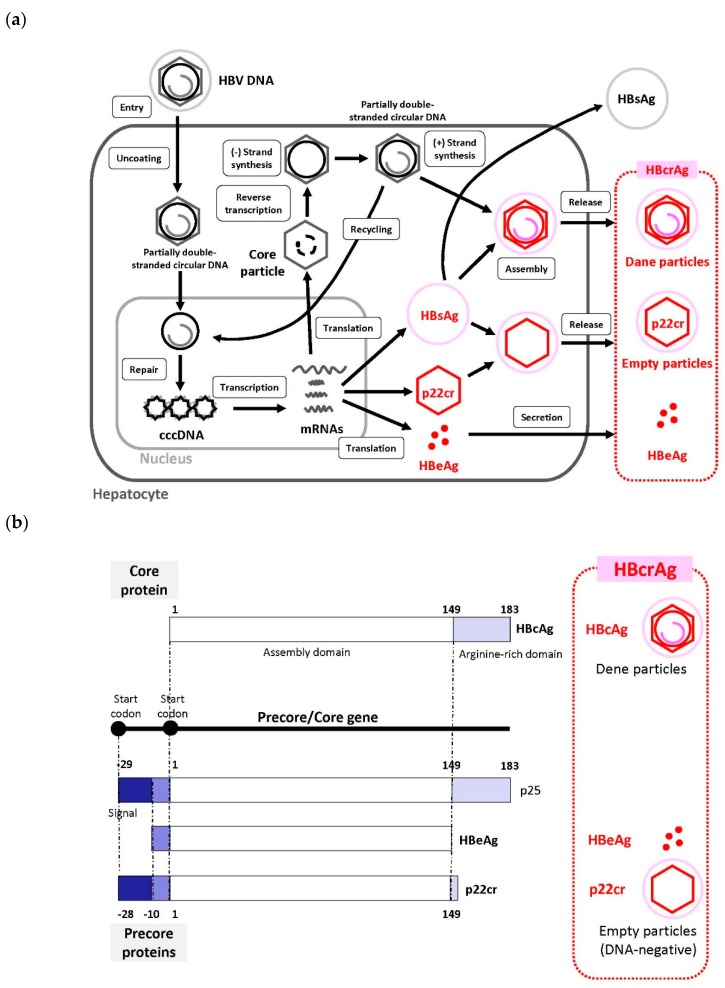
Schematic representation of the Hepatitis B virus (HBV) lifecycle, from HBV entry into hepatocytes to release from hepatocytes. (**a**) The c covalently closed circular DNA (ccDNA) is present as minichromosomes of 5 to 50 in one hepatocyte. A portion of the incompletely circularized double stranded DNA, which has been reverse transcribed and synthesized, is transferred again into the nucleus. It is recycled as cccDNA to maintain the stock amount of cccDNA [6]. HBcrAg is produced from cccDNA. (**b**) Hepatitis B core-related antigen (HBcrAg) is a denatured mixture consisting of hepatitis B envelope antigen (HBeAg), hepatitis B core antigen (HBcAg), and 22-kDa precore protein (p22cr) coded with the precore/core region [32,33,34]. HBeAg: Information about replication status of the virus; p22cr: Empty virus particle; HBcAg: Undetectable protein in blood because it is always part of a complex. These three different types of antigen proteins are translated by the mRNA of transcriptional products of cccDNA, which itself is generated by the HBV replication process in hepatocytes [36]. By serologic testing, HBeAg, p22cr, and HBcAg can be measured as HBcrAg all together [33,35]. Abbreviations: HBV, hepatitis B virus; cccDNA, covalently closed circular DNA; HBcrAg, hepatitis B core-related antigen; HBeAg, hepatitis B envelope antigen; HBcAg, hepatitis B core antigen; p22cr, 22-kDa precore protein.

We recently reviewed the HBV life cycle [6], which represents the period from the entry of HBV into hepatocytes to its release from hepatocytes. Recently, sodium taurocholate cotransporting polypeptide (NTCP) was identified as a receptor for HBV entry, which enabled the establishment of a susceptible cell line that efficiently supports HBV infection [26,27]. In this section, we provide brief information regarding the HBV life cycle (Figure 1a).

HBV (Dane particle) enters hepatocytes by primary attachment of the pre-S1 region on the virion envelope to the NTCP receptor [27,28,29] and possible extra hepatocyte-specific factors on the cell surface. The HBV envelope fuses with the cell membrane of the host hepatocyte, and the virion is endocytosed [6]. Then, the virion is uncoated and transported into the nucleus. The HBV membrane releases the viral DNA (partially double-stranded circular DNA) with a core particle into the cytoplasm [29]. The viral membrane is lost (uncoating) and the viral nucleocapsid containing the viral genomic DNA is transported into the nucleus in its relaxed circular form. This viral relaxed circular DNA or linear DNA genome, with a protein attached to the 5′ end of the minus strand and a short RNA attached to the 5′ end of the plus strand [30], is converted into cccDNA through covalent ligation. In the nucleus, fully double-stranded DNA synthesis is completed by viral DNA polymerase and converted to cccDNA [28,29]. cccDNA formation is most likely formed via the DNA repair mechanism; however, this remains poorly understood [6,28]. cccDNA is responsible for viral persistence and is highly resistant to antiviral therapy. It serves as the template for the transcription of viral mRNAs.

Pregenome mRNA is involved in the synthesis of core protein (nucleocapsid subunit) and viral reverse transcriptase. The viral genome is replicated by the reverse transcription of pregenomic RNA. During this process, both the protein and the RNA are removed [30]. Reverse transcriptase binds to the 5′ end of its own mRNA template, and the complex is then packaged into nucleocapsids, where viral DNA synthesis occurs. cccDNA is transcribed into the pregenomic and subgenomic mRNAs by host RNA polymerase [6,28,29].

After the synthesis of (-) and (+) strand DNAs, the nucleocapsid, containing the partially double-stranded circular DNA, is generated. These nucleocapsids can also move into the nucleus to increase the copy numbers of cccDNA. Because cccDNA does not undergo semiconservative replication, all cccDNA copies result from viral DNA made in the cytoplasm via the reverse transcription pathway [31]. HBsAg and the nucleocapsid containing partially double-stranded circular DNA are assembled to generate a new complete virion [29]. An increase in the level of viral envelope proteins inhibits the synthesis of high levels of cccDNA, which can be toxic to hepatocytes. Mature nucleocapsids may be secreted as mature HBVs (Dane particles) through exocytosis to infect other hepatocytes. Mature nucleocapsids are then recycled back into the nucleus to amplify the cccDNA [6,27,28,30].

## 3. HBcrAg and Its Measurement

### 3.1. What Is HBcrAg?

HBcrAg consists of three proteins coded by the precore/core region including HBeAg, hepatitis B core antigen (HBcAg) that mainly consists of a Dane particle, and a 22-kDa precore protein (p22cr), which is an HBV DNA-negative (empty) particle [32,33,34]. By serologic testing, HBeAg, p22cr, and HBcAg can all be measured as HBcrAg [33,35].

HBeAg is a circulating peptide derived from the core gene, which is then transformed and secreted from hepatocytes. HBcAg is an inner nucleocapsid surrounding the viral DNA. p22cr is present in HBV DNA-negative and HBcAg-deficient Dane-like particles [36]. HBeAg, HBcAg, and p22cr are all products of the precore/core gene and share an identical 149 amino acid sequence (Figure 1b) [36,37]. Both HBcAg and HBeAg are the target of cytotoxic T-cells. Thus, HBcrAg, which consists of three products of the precore/core gene, can induce host cellular immune responses [38].

### 3.2. HBcrAg Measurement

The first report of HBcrAg in 2002 was related to the development of a sensitive enzyme immunoassay specific for HBcAg and HBeAg [32]. Kimura et al. designated the precore/core gene products, comprised of HBcAg and HBeAg, as HBcrAg. The sera were pretreated to inactivate antibodies and to denature antigen before the assay. This assay detects HBcAg and HBeAg even in anti-HBc or anti-HBe positive specimens. On HBeAg/anti-HBe seroconversion panels, the HBcrAg concentration changes according to HBV DNA levels. Because the HBcrAg concentration reflects the HBV DNA level, it can be used supplementary to HBV DNA for the monitoring of CHB patients [32].

With the development of the HBcrAg assay, the clinical performance of a newly developed CLEIA for the detection of HBcrAg in CHB patients was described [33]. In that report, the HBcrAg concentration correlated linearly with the HBV DNA concentration (*p* < 0.001) over a range that varied 100,000-fold. The accuracy of the measurement of HBV load obtained by the HBcrAg assay was not affected by the absence of HBeAg in sera or the presence of precore mutations in the HBV genome. More detailed information of the latest HBcrAg measurement was described in a previous review by Mak et al. [39].

## 4. Correlation between HBcrAg and Other HBV Markers

### 4.1. Serum or Intrahepatic HBV DNA Levels

In the first report of HBcrAg, Kimura et al. demonstrated that the HBcrAg concentration changed according to HBV DNA levels. They proposed that the HBcrAg assay was a simple method for monitoring CHB patients [32]. Since then, the significance of HBcrAg measurement to monitor CHB has been suggested by several studies demonstrating that serum HBcrAg concentrations have a good correlation with HBV DNA levels [33,40,41,42]. The studies reporting a correlation between serum HBcrAg and HBV DNA levels are summarized in Table 1.

Serum HBcrAg directly reflects the serum HBV DNA concentration, regardless of HBeAg status (Table 1a) [33,40,41,42]. Intrahepatic total HBV DNA also reflects the serum HBcrAg level in patients with and without treatment (Table 1b) [40,42].

### 4.2. Intrahepatic cccDNA Levels

In a pioneering study, serum cccDNA levels were reported to correlate positively with intrahepatic cccDNA (*r* = 0.481, *p* = 0.002), although the correlation was not strong [43]. Other studies have reported that serum HBcrAg levels are closely associated with intrahepatic cccDNA levels in addition to serum HBV DNA [34,40,44,45,46]. Therefore, serum HBcrAg levels correlate with HBV virological markers [40]. Several reports describing the relationship between HBcrAg and cccDNA levels are summarized in Table 1. Two studies with a large number of patients reported a correlation coefficient of approximately 0.7 (Table 1c) [40,42].

In comparison, serum HBV DNA also correlated well with intrahepatic cccDNA (correlation coefficient 0.7, *p* < 0.001) [42]. However, although patients with anti-viral therapy often have undetectable serum HBV DNA, 78% of these patients still had detectable serum HBcrAg [42]. Therefore, in the context of serum DNA undetectability, HBcrAg would be the preferred serum marker to estimate the quantity of intrahepatic cccDNA.

Hasegawa et al. created a unique prediction model for the measurement of cccDNA levels in CHB patients and confirmed the model’s predictive accuracy [47]. By multivariate analysis for the prediction of cccDNA levels in patients without previous nucleos(t)ide analogue (NA) therapy, fasting blood sugar (FBS) (*p* = 0.0227), HBeAg (*p* = 0.0067), and log10 HBsAg (*p* = 0.0497) were significant, whereas HBcrAg only showed a trend toward significance (*p* = 0.0562). A formula was constructed and named the FBS-cres score based on the variables used (FBS, HBcrAg, HBeAg, and HBsAg). The FBS-cres score was calculated as: 3.1686 − (0.0148 × FBS) + (0.1982 × HBcrAg) + (0.0008168 × HBeAg) + (0.1761 × log10 (HBsAg)). In patients without previous nucleos(t)ide analogues (NA) therapy, a significant correlation was noted between HBcrAg and cccDNA levels (*p* < 0.0001), while the FBS-cres score was more closely correlated to cccDNA level (*p* < 0.0001) [47].

### 4.3. HBsAg

In a 2014 study, among the whole patient cohort (*n* = 404), there was a strong correlation between HBsAg-HQ levels, HBV DNA, HBsAg and HBcrAg levels (*r* = 0.762, 0.804, and 0.818, respectively, all *p* < 0.001). Serum HBcrAg levels also correlated strongly with serum HBV DNA and HBsAg levels (*r* = 0.854 and 0.703, respectively, both *p* < 0.001) [25]. These results suggest that HBcrAg correlates more with HBsAg-HQ, which is a more sensitive assay, than with the conventional HBsAg assay.

### 4.4. HBV RNA

The replication cycle of HBV DNA starts with the endonuclear cccDNA transcription of pre-genomic RNA (pgRNA). pgRNA is enveloped in the nucleocapsid during the creation of the virus, and HBV DNA polymerase transcribes offspring DNA using pgRNA as the template. The offspring DNAs are then recycled in the nucleus to mediate viral persistence. Some offspring DNAs are assembled into complete virions in the endoplasmic reticulum and secreted from hepatocytes [48,49,50].

Recently, a non-invasive technique was reported where circulating HBV RNA might be used as a new serum biomarker for HBV infection, treatment, and prognosis [51,52]. Serum HBV RNA undetectability usually occurs prior to HBcrAg undetectability [53]. Huang et al. [54] further examined the correlation between serum HBV RNA and intrahepatic cccDNA levels [54] and found that serum HBV RNA reflected cccDNA levels in HBeAg-positive CHB patients, and total serum nucleic acids (HBV DNA plus RNA) better reflected intrahepatic cccDNA levels compared with serum HBV RNA or HBV DNA levels [50].

Although serum HBV RNA and HBsAg levels differ significantly between HBeAg-positive and HBeAg-negative patients, serum HBcrAg correlates with intrahepatic cccDNA levels better than HBV RNA and HBsAg, regardless of HBeAg status. Similarly, Chen et al. assessed the correlation of serum HBcrAg with HBV RNA and HBsAg and investigated whether serum HBcrAg was superior to serum HBV RNA and HBsAg as an indicator of intrahepatic cccDNA in HBeAg-positive and HBeAg-negative CHB patients. They reported that serum HBcrAg was better correlated with intrahepatic cccDNA than serum HBV RNA and HBsAg, irrespective of HBeAg status [55].

### 4.5. HBV Gene Deletion

In the preS1/S2 region of the HBV genome, deletions are commonly observed in association with CHB advancement [56]. Considering this background, quantification of the preS mutations might improve our understanding of the mechanism of CHB progression [34].

Importantly, preS codon 132–141 deletions have a strong influence on the HBV life cycle and pathogenesis [56]. Suzuki et al. examined the preS1/S2 regions of HBV in 90 CHB patients without antiviral therapy by deep sequencing, and which deleted regions influenced by viral markers. Deletion frequency analysis for each CHB patient indicated that deletions occurred most frequently in the preS2 codon 132–141 region. Serum HBcrAg, fibrosis 4 index (FIB-4 index), HBV DNA, and preS1/S2 start codon mutations were significantly associated with the deletion (*p* < 0.01). They concluded that preS codon 132–141 deletions have a significant influence on clinical characteristics and viral markers, even when they exist as a minor population [56].

## 5. CHB Natural History Including HBeAg Seroconversion

Serum HBcrAg is also a useful marker for disease monitoring to predict the clinical outcome of CHB [39,57]. In this section, we provide information regarding the efficacy of serum HBcrAg to predict CHB natural history.

### 5.1. HBcrAg in Different Stages of Chronic Infection

An initial report by Misawa et al. described the efficacy of HBcrAg measurement in monitoring CHB patients before and after seroconversion with additional information about patient virological characteristics [58]. The HBV DNA level was similar between the active replication and inactive replication groups, although the HBcrAg level was significantly lower in the active replication group than in the inactive replication group before seroconversion. The levels of HBV DNA and HBcrAg were markedly decreased around the time of seroconversion in the inactive replication group, while these levels did not change or were decreased slightly in the active replication group. Because serum HBcrAg levels mainly reflect serum HBeAg levels, a lower HBcrAg level observed after seroconversion in both groups may contribute to seroconversion [58].

Considering the natural history of CHB, HBcrAg levels change significantly in relation to the disease phases of CHB, as described by two reports of treatment-naïve CHB patients from Asia (*n* = 404) and Europe (*n* = 249) [25,35]. In both reports, serum HBcrAg levels differed significantly between HBeAg-positive and -negative CHB patients. Overall, HBeAg-positive patients have a higher HBcrAg level compared with HBeAg-negative patients (Table 2). This phenomenon is associated with the decreased production of HBeAg after HBeAg seroconversion. Specifically, in HBeAg-positive CHB patients, the HBcrAg levels were 8.54 and 7.92 log U/mL in the immune tolerance phase and immune clearance phase, respectively (both *p* < 0.001) [25]. These results suggest that HBeAg-positive CHB patients with lower HBcrAg levels are more likely to be under a stronger immune control.

### 5.2. Prediction of HBeAg Seroconversion

Similar results were reported by Testoni et al. [59]. Serum HBcrAg levels were significantly higher in HBeAg-positive patients than in HBeAg-negative patients without antiviral treatment and they correlated with serum HBV DNA, intrahepatic HBV DNA, pgRNA and cccDNA levels, as well as transcriptional activity. Patients who were negative for HBcrAg (<3 log U/mL) had lower amounts of intrahepatic cccDNA and lower cccDNA activity than HBcrAg-positive patients. Higher HBcrAg levels were associated with higher serum HBV DNA, intrahepatic HBV DNA, pgRNA, cccDNA transcriptional activity, and fibrosis and necroinflammatory activity scores [59].

For HBeAg-negative patients, the HBcrAg levels were significantly lower in the HBeAg-negative inactive carrier phase than in the HBeAg-negative active phase (2.60 vs. 4.92 log U/mL, respectively; *p* < 0.001) (Table 2) [25]. A higher amount of HBcrAg in the HBeAg-negative active phase compared with the HBeAg-negative inactive carrier phase was associated with significant necroinflammatory activity and fibrosis [60].

### 5.3. Prediction of HBsAg Seroconversion

An advanced study by Loggi et al. evaluated serum HBcrAg levels in HBeAg-negative CHB patients, and its ability to determine the clinical profile, in comparison with serum HBsAg levels [57]. In an overall population of 160 HBeAg-negative patients, HBcrAg levels were significantly higher in 85 CHB patients relative to 75 clinically inactive carriers. A value of 2.5 log U/mL produced the optimal cut-off required to identify clinically inactive carriers, with diagnostic accuracy comparable to serum HBsAg levels. By long-term clinical evaluation, a single measurement of HBcrAg at the established cut-off was optimally consistent with clinical outcome [57].

For patients with spontaneous HBsAg seroclearance, most (79%) had undetectable HBcrAg levels, suggesting a more quiescent disease state. Of the 21% patients who still had detectable serum HBcrAg, the median HBcrAg level was 2.7 log U/mL [25,61]. These findings suggest a potential use of HBcrAg to further define the disease phases of CHB, although the optimal cut-offs remain to be determined [39]. 

## 6. Anti-HBV Treatment

Recent advances in the development of NAs have made it possible to decrease hepatitis activity and to suppress serum HBV DNA [56]. However, there remains an unmet need for convenient biomarkers to evaluate the occurrence risk of NA resistance, and the appropriateness of discontinuing NAs in patients with CHB. From lamivudine (LAM) as a classical NA to tenofovir (TDF) as a novel NA, this section will discuss clinical reports of risk prediction related to NA treatment.

All patients with HBeAg-positive or -negative CHB, defined by HBV DNA >20,000 or >2000 IU/mL, ALT > upper limit of normal, and/or at least moderate liver necroinflammation or fibrosis, should be treated [11].

In HBeAg-positive CHB patients, treatment-induced HBeAg loss and seroconversion to anti-HBe is a valuable endpoint. Once seroconversion to anti-HBe occurs, the reduction of HBcrAg and HBsAg followed by the achievement of HBsAg loss is widely referred to as a major goal of current treatment efforts in CHB [11].

In HBeAg-negative CHB patients, the goal of antiviral therapy is a sustained off-treatment response and HBsAg clearance [62]. However, because it is hard to obtain HBsAg clearance during NA therapy, the reduction of HBsAg as well as HBcrAg might be useful when judging the efficacy of NA therapy.

Hence, HBcrAg could be more useful in the HBeAg-negative phase. For example, the JSH Guidelines for the Management of HBV Infection [63] described the criteria for the cessation of NA therapy (see Table 15, [63]). The three laboratory criteria for the cessation of NA therapy are: (1) At least two years of administration of NAs; (2) undetectable serum HBV DNA levels (using real time PCR); and (3) negative serum HBeAg at the time of treatment cessation. When the above criteria are met, it is possible to predict the risk of relapse from HBsAg and HBcrAg levels at the time of cessation of therapy. NA therapy should be continued in the high-risk group as described in Table 15 “Risk of relapse following cessation of NA therapy” [63].

### 6.1. Change in HBcrAg and Other HBV Markers under NA Therapy

In a study of 43 patients treated with NA (median; 126 months), 98% had undetectable serum HBV DNA, although 51% still had detectable intrahepatic cccDNA [64]. Similar findings were reported in 24 patients treated with sequential therapy of pegylated interferon (PEG-IFN) and adefovir (ADV) followed by ADV monotherapy—46% and 66% patients had undetectable serum HBV DNA and detectable intrahepatic cccDNA, respectively [65]. Consequently, a reduction in serum HBV DNA does not correlate with a reduction of intrahepatic cccDNA for those receiving anti-HBV therapy.

More notably, the reduction in HBcrAg demonstrated a good correlation with the magnitude of change in intrahepatic cccDNA levels [40,42]. In contrast to serum HBV DNA, the reduction of HBcrAg was slower during NA treatment, with an increase in the ratio of serum HBcrAg:HBV DNA after three months of LAM [33]. The difference between serum HBcrAg and HBV DNA can be explained by the action of NA on reverse transcription and the subsequent prevention of HBV DNA replication, while HBcrAg production remains natural. Therefore, it is not surprising that in patients receiving NA treatment with undetectable serum HBV DNA, 78% had persistent HBcrAg [42]. Even in patients with documented HBsAg seroclearance, 21% had detectable serum HBcrAg, in contrast to detectable serum HBV DNA in only 2.1% [34,61]. For PEG-IFN treatment, two studies exploring the reduction of cccDNA and HBcrAg reported conflicting results. While one study showed a similar and significant decline of serum HBcrAg levels and cccDNA [66], the other study did not find a significant reduction of serum HBcrAg with PEG-IFN [67]. The number of patients in these two studies were too low (*n* = 58 and 8, respectively) to demonstrate any specific pattern of HBcrAg levels during PEG-IFN therapy.

Serum HBcrAg levels at baseline and changes while on anti-HBV therapy may also predict suitable indicators in CHB patients. For patients treated with PEG-IFN, a baseline high HBcrAg level >8 log U/mL conferred a >94.4% negative predictive value (NPV) for achieving HBeAg seroconversion and suppression of HBV DNA at 12 weeks (*n* = 46) [62]. In another study, 50 patients treated with sequential therapy of PEG-IFN plus NA for 4 weeks followed by PEG-IFN for 20 weeks reported that a high HBcrAg level (>4.5 log U/mL) at the beginning of therapy predicted no response and no HBeAg seroconversion at 24 months after finishing treatment [68]. Furthermore, HBcrAg levels changed by the treatment might predict clinical outcomes. In 58 patients treated with PEG-IFN, the HBcrAg level at week 12 predicted HBeAg seroconversion at 24 weeks after finishing treatment with an area under the curve of the receiver operating characteristic (AUROC) of 0.896 [66]. For patients treated with NA therapy (*n* = 39), the HBcrAg levels were lower in patients with NA-induced HBeAg seroconversion compared with patients who remained HBeAg-positive [40]. Studies also reported the use of HBcrAg in predicting HBsAg seroclearance, although the findings remain inconclusive. The AUROC for HBsAg loss was 0.763 if the mean baseline HBcrAg plus HBsAg were used to predict HBsAg loss, although this performed no better than using HBsAg titers alone (AUROC 0.771) [69]. Therefore, HBcrAg may not be more valuable than the HBsAg level in terms of predicting HBsAg loss in treatment-experienced patients.

HBcrAg measurement is valuable for identifying patients at low risk of LAM resistance [45]. Tanaka et al. compared the clinical usefulness of HBcrAg with that of serum HBV DNA assay in predicting the occurrence of LAM resistance in 81 CHB patients. Twenty-five patients (31%) developed LAM resistance during the follow-up period (median; 19.3 months). HBcrAg and HBV DNA levels decreased after the initiation of LAM administration, although the HBcrAg level decreased significantly more gradually than the HBV DNA level. LAM resistance did not occur during the follow-up period in 19 patients with a HBcrAg level <4.6 log U/mL at six months of treatment, although it did occur in 50% of the remaining patients within two years [45].

As an advanced technique, a combination of HBcrAg and HBsAg assays may be used to identify patients who are unlikely to achieve treatment end-points, i.e., HBeAg seroconversion [70]. Wang et al. identified predictors of seroconversion using serum quantitative HBsAg and HBcrAg, in HBeAg-positive patients. Data and samples were obtained from 118 HBeAg-positive adults with HBV genotypes A-G treated with NA. About 36.4% of patients achieved HBeAg seroconversion after NA treatment (median; 39 months). Regarding the treatment kinetics of HBV DNA, HBsAg and HBcrAg differed between patients with and without HBeAg seroconversion [70].

The baseline HBcrAg level is an independent predictor of long-term HBcrAg below the limit of detection [71]. Wang et al. investigated the long-term kinetics of serum HBcrAg and its correlation with serum HBsAg in CHB patients with NA therapy over 8 years. Among 94 patients, serum HBcrAg gradually decreased from baseline to year 8 in HBeAg-negative and -positive patients. At eight years from the start of NA treatment, 21.3% of patients achieved a serum HBcrAg level of <3 log10 U/mL, and only baseline HBcrAg was an independent predictor [71].

### 6.2. NA Cessation Point

Although most patients treated with NA will continue therapy, some may select to discontinue therapy. The decision of NA cessation has been traditionally based on viral serological markers, serum HBV DNA, and alanine transaminase (ALT) and more recently, on serum HBsAg levels. A decrease in HBcrAg was observed under NA therapy, and the pattern of reduction may provide predictive information on the risk of post-treatment HBV reactivation [67]. A serum HBcrAg level >3.7 log IU/mL at NA cessation predicted virological relapse within one year of NA cessation [72]. A similar report described results for 34 CHB patients treated with LAM, where high HBcrAg levels (median; 4.9 log U/mL) at NA cessation predicted relapse regardless of undetectable HBV DNA for at least six months [73]. Therefore, serum HBcrAg as a surrogate marker may have a better influence on patients who are planning NA cessation.

Conversely, an HBcrAg level at LAM cessation point <3.4 log U/mL was the only independent predictive factor without relapse after NA cessation. Shinkai et al. examined which factors predicted ALT flares after LAM cessation [74]. Twenty-two Japanese CHB patients, in whom LAM was stopped after serum HBV DNA had remained undetectable for at least the six following months, were examined. After LAM cessation, 11 patients (50%) had serum ALT > 80 U/L (relapsers), and the remaining 11 (50%) were non-relapsers in the follow-up period (median; 28 months). A higher HBcrAg level at LAM cessation (relapsers; 4.5 ± 1.0 log U/mL versus non-relapsers; 3.4 ± 0.9 log U/mL, *p* = 0.0145) was a significant relapse predictor. All patients with HBcrAg <3.0 log U/mL at cessation had no ALT flares [74].

Regarding other NAs such as entecavir (ETV) or TDF, the HBcrAg level at NA cessation is an independent relapse predictor, as well as HBsAg, age, ALT, and TDF use. Hsu et al. enrolled 135 CHB patients who had stopped ETV or TDF after accomplishing viral reduction for a median of 25.2 months. All patients stopped NA with negative HBeAg and undetectable HBV DNA. During the follow-up period (median; 25.9 months), clinical relapse and HBsAg loss occurred in 66 and 8 patients, respectively, with a five-year cumulative incidence of 56.1% and 8.8%, respectively. A score (SCALE-B) was calculated using the equation 35 × HBsAg (log IU/mL) + 20 × HBcrAg (log U/mL) + 2 × age (year) + ALT (U/L) + 40 for TDF use. The concordance rates for clinical relapse were 0.87, 0.88, 0.87, 0.85, and 0.90 at one, two, three, four, and five years, respectively. Moreover, HBsAg loss occurred completely in low-risk patients predicted by the score [75].

### 6.3. HBcrAg and HBV RNA Related to NA Treatment

As a non-invasive technique, measurement of serum HBV RNA levels may serve as a new serum biomarker for HBV infection, treatment, and prognosis [52]. Monitoring of serum HBV RNA and HBcrAg levels is suitable for patients with undetectable HBV DNA under NA treatment.

Liao et al. evaluated the clinical significance of serum HBV RNA, HBcrAg, and hepatitis B core antibody (anti-HBc) levels in CHB patients with undetectable HBV DNA during NA treatment. They concluded that these factors gradually decreased with time under NA treatment [53]. Fifty-seven patients who received continuing NA treatment for a median 5.83 years (25%, 75% percentiles; 4.67, 7.75) were enrolled. The HBV RNA level significantly correlated with HBcrAg (*r* = 0.629; *p* < 0.001), but not HBsAg levels (*p* = 0.1460). However, the HBcrAg level significantly correlated with the HBsAg level (*r* = 0.469; *p* < 0.001), and HBeAg-positive samples had higher HBV RNA, HBcrAg, and HBsAg levels compared with HBeAg-negative samples (all *p* < 0.05) [53].

## 7. HCC Development

### 7.1. HCC Occurrence

It is recognized that HCC may develop in a large number of patients, even after the introduction of NA treatment, and the prediction of which patients will develop liver disease including HCC after NA introduction is difficult [56]. Several HBV markers have been identified as factors associated with the development of HCC in patients with CHB. Baseline HBV DNA is a known independent predictor of HCC [76]. Recent studies revealed that serum HBcrAg level was associated with the development of HCC in patients [77,78,79].

For treatment-naïve patients, HBcrAg was superior to HBV DNA in terms of predictive power for HCC development in a large cohort study [79]. During the follow-up period (median; 10.7 years), 78 of 1031 CHB patients (7.6%) without NA developed HCC. A Cox proportional hazard model using the covariates of HBV genotype status, HBV DNA levels, HBcrAg levels, HBeAg status, and basal core promotor (BCP) status indicated that HBcrAg > 2.9 log IU/mL (hazard ratio [HR], 5.05; 95% confidence interval [CI], 2.40–10.63) and BCP mutation (HR, 28.85; 95% CI, 4.00–208.20) were independently associated with the incidence of HCC [79]. In another report, serum HBcrAg provided a good method for monitoring cccDNA in HCC and was a good prognostic predictor for HCC patients [80]. Serum HBcrAg correlated positively with HBV DNA regardless of HBeAg status. From liver specimens provided by 89 HCC patients, both HBcrAg and HBV DNA were associated with cccDNA in patients with higher serum HBV DNA (>4 log IU/mL). In patients with lower HBV DNA (≤4 log IU/mL), no relationship between HBV DNA and cccDNA was observed [80].

For treatment-experienced patients, NA reduced but could not eliminate the risk of HCC [81], and HBcrAg positivity after NA for at least a two-year duration was an independent risk factor for HCC [78]. In 76 CHB patients with undetectable serum HBV DNA treated with NA, the pre-treatment HBcrAg levels were significantly higher in the HCC group compared with the matched control group (5.45 log U/mL vs. 4.55 log U/mL respectively; *p* = 0.005) and a pre-treatment cut-off of 4.67 log U/mL independently predicted HCC. Furthermore, a post-treatment HBcrAg > 3.89 log U/mL predicted HCC with an odds ratio of 3.27. When only noncirrhotic patients were considered, a cut-off of >3.90 log U/mL predicted HCC with an odds ratio of 5.95 [82]. Similarly, patients with persistently high HBcrAg levels regardless of NA treatment were more likely to develop HCC despite sustained viral suppression associated with continuing NA treatment [76]. Hosaka et al. investigated whether the baseline and on-treatment serum HBcrAg levels could predict HCC incidence in 1268 CHB patients with NA therapy for >1 year. During the follow-up period (median; 8.9 years), 113 patients (8.9%) developed HCC (10.3/1000 person-years). Patients with persistently high on-treatment HBcrAg levels had a higher risk of HCC than those with low HBcrAg levels (HBeAg-positive: HR, 6.15, 95% CI: 1.89–20.0, *p* = 0.003; HBeAg-negative cohort: HR, 2.54, 95% CI: 1.40–4.60; *p* = 0.002) in a multivariate Cox regression analysis [76]. To investigate the long-term effect of NA therapy on HCC progression, Kumada et al. compared CHB patients who received NA therapy with those who did not [77]. In the follow-up period, HCC developed in 57 of 234 patients (24.4%). Factors significantly associated with HCC incidence included higher age (HR, 4.36 (95% CI, 1.33–14.29), *p* = 0.015), NA treatment (0.28 (0.13–0.62), *p* = 0.002), BCP mutations (12.74 (1.74–93.11), *p* = 0.012), high HBcrAg (2.77 (1.07–7.17), *p* = 0.036), and high gamma glutamyl transpeptidase levels (2.76 (1.49–5.12), *p* = 0.001). They concluded that higher serum levels of HBcrAg and BCP mutations were associated with progression to HCC, independent of NA therapy [77].

Modulating HBV transcriptional factors by metformin in combination with NA therapy potentiated anti-HBV activity and reduced the incidence of HCC in HBcrAg-positive patients [78]. Honda et al. showed that age >60 years (HR; 2.66), FIB-4 index >2.1 (HR; 2.57), and the presence of HBcrAg (HR; 3.53) during NA treatment were significantly associated with the development of HCC. The amount of HBV DNA and pregenomic RNA in the liver was significantly higher in 16 HBcrAg-positive patients, compared with 12 HBcrAg-negative patients, suggesting active HBV replication in HBcrAg-positive livers. Hepatic gene expression profiling showed that HBV-promoting transcriptional factors, including hepatocyte nuclear factor-4α, peroxisome proliferator-activated receptor α, and liver receptor homolog-1, were upregulated in HBcrAg-positive livers. However, the overexpression of precore/core in HepG2 cells increased the levels of these transcriptional factors. Metformin efficiently repressed HBV replication in primary human hepatocytes [78].

A recent report showed that a combination of HBsAg and HBcrAg values was an excellent biomarker for assessing HCC risk in CHB patients [83]. Four hundred and forty-nine consecutive patients with CHB were included in the study and the association of HBsAg and HBcrAg with HCC risk was investigated cross-sectionally, as well as longitudinally. When the high cut-off values of HBsAg and HBcrAg were defined as 3.0 log IU/mL and 3.0 log U/mL, respectively, patients with HCC history were frequently found in the low HBsAg (*p* = 0.002) and high HBcrAg groups (*p* < 0.001). When HBsAg and HBcrAg were combined, a history of HCC was most frequent in the subset with low HBsAg and high HBcrAg, among HBeAg-negative patients, regardless of NA therapy. In a longitudinal analysis of the subsequent development of HCC in 338 patients without HCC history at enrollment, HCC developed significantly more frequently in the low HBsAg/high HBcrAg group (*p* = 0.005) [83].

### 7.2. HCC Recurrence

Serological markers have also been used to predict HCC recurrence after resection or radio-frequency ablation. However, post-surgical HCC recurrence rates remained high regardless of the usage of NA, with reported recurrence rates of up to 41.8% over two years [84]. Several factors are associated with higher recurrence, including a pre-operative serum HBsAg titer >1000 IU/mL, baseline HBeAg positivity, presence of cirrhosis, tumor size, tumor number, macrovascular invasion, and the usage of NAs other than ETV or TDF [85,86].

Recently, studies have also confirmed the predictive value of HBcrAg in HCC recurrence after curative surgery. In a study of 55 patients, serum HBcrAg level >4.8 log U/mL at the time of HCC diagnosis gave an HR of 8.96 for subsequent HCC recurrence within two years [76]. In another report of 21 HCC patients who experienced liver transplantation, five patients developed HCC recurrence after liver transplantation (2/14 HBcrAg-positive patients and 3/7 HBcrAg-negative patients). Nevertheless, the positivity for serum HBcrAg after liver transplantation did not show a significant correlation with the risk of HCC recurrence [87]. Therefore, serum HBcrAg level before operation might be a potential marker to stratify post-surgical surveillance approaches and to identify patients with a high risk of recurrence.

Finally, the HCC recurrence-free survival rates were significantly lower in HCC patients with high intrahepatic cccDNA and serum HBcrAg levels than those with low cccDNA/HBcrAg levels (*p* = 0.035, *p* = 0.003, respectively) [80].

## 8. HBV Reactivation

The association between HBcrAg and HBV reactivation in 124 HBsAg-negative and anti-HBc-positive patients undergoing high-risk immunosuppressive therapy (rituximab, *n* = 62; allogeneic hematopoietic stem cell transplantation, *n* = 62) was investigated [88]. HBV reactivation occurred in 31 patients, with a two-year cumulative reactivation rate of 40.4%. Serum HBcrAg was detected in 43 (34.7%) patients. Baseline HBcrAg positivity was significantly associated with HBV reactivation (*p* = 0.004). HBcrAg-positive patients had a significantly higher two-year HBV reactivation rate than HBcrAg-negative patients (71.8% vs. 31%, *p* = 0.002). Therefore, serum HBcrAg positivity might play a role in identifying patients who will benefit from prophylactic NA treatment [88].

## 9. HBV Reinfection after Liver Transplantation

HBV re-infection after liver transplantation can be almost completely repressed by NA treatment and hepatitis B immunoglobulins [89,90]. However, there is no indicator of HBV replication because test results for serum HBsAg and HBV DNA are negative after transplantation [91]. HBcrAg was also suggested to be a marker of HBV reinfection after liver transplantation [91]. In this section, we discuss several reports of the relationship between liver transplantation and HBcrAg.

The first report describing HBV biomarkers related to liver transplantation was published in Japan in 2009 [92]. Fujimoto et al. proposed the potential use of HBcrAg and discussed the dynamics of HBV in 12 patients after HBV-related living donor liver transplantation. In the period before operation, all cases were negative for serum HBV DNA and HBsAg under prophylaxis therapy. In the period after operation, 5/12 patients had positive serum HBcrAg, and when in a stable state, 6 had positive serum HBcrAg. The mean levels of HBcrAg after HBV-related living donor liver transplantation were significantly lower than those seen in the period before operation [92]. Therefore, HBcrAg is a suitable marker of HBV replication in the post transplantation stage.

Yasunaka et al. investigated intrahepatic HBV reinfection status using their clinically successful protocol [93]. No patients showed recurrence of serum HBsAg or HBV DNA. However, serum HBV DNA, cccDNA, and HBcrAg were positive in 57% and 48% of patients after liver transplantation, respectively. High serum HBV DNA (>3 log10 copies/mL) and HBcrAg (> 4 log10 IU/mL) levels, were associated with high levels of cccDNA after liver transplantation. Patients with high serum HBV DNA and HBcrAg levels before liver transplantation should be followed with special care for HBV recurrence [93]. Another study reported that maintaining patient serum HBcrAg and cccDNA negative after liver transplantation might contribute to long-term graft survival [91]. Matsuzaki et al. examined serum HBcrAg and intrahepatic cccDNA levels in 20 patients after liver transplantation. Serum HBcrAg and cccDNA levels were significantly correlated with each other (*r* = 0.616, *p* < 0.001). From a clinical aspect, the fibrosis stage was significantly lower in both HBcrAg- and cccDNA-negative patients than in HBcrAg- or cccDNA-positive patients [91]. The clinical applications of HBcrAg are summarized in Table 3.

## 10. Upcoming Anti-HBV Strategies

Although effective treatments such as IFN-based regimens and NA have been developed, they are not perfect because cccDNA remains in hepatocytes. To date, no therapies have been able to eradicate HBV from host cells. Consequently, regular monitoring of responses during and after treatment is required [94]. In this section, new potential therapeutic agents, especially related to their effect on intrahepatic cccDNA, are described. As we described in the “Intrahepatic cccDNA levels” subsection (Section 4.2), HBcrAg is the preferred serum marker to estimate the quantity of intrahepatic cccDNA. Therefore, HBcrAg will be used as a surrogate marker of serum HBV DNA when administering these upcoming anti-HBV treatments.

In HBeAg-negative CHB patients with NA treatment, it is important to monitor HBcrAg because HBV DNA is undetectable after NA treatment. However, because the present HBcrAg assay has a low sensitivity, a novel HBcrAg assay with approximately 10-fold higher sensitivity is being developed. Furthermore, monitoring using a higher sensitivity HBcrAg assay will be useful to judge therapeutic effects because capsid assembly modifiers (CAMs), which may be a new potential therapeutic agent, can directly inhibit the intrahepatic core protein and possibly cccDNA as well.

### 10.1. Capsid Assembly Modifier

One of the most promising viral targets in current anti-HBV drug development is the core protein, because of its multiple roles in the viral life cycle [95]. Corcuera et al. examined differences in the mode of action and anti-HBV activity of representatives of six different CAM scaffolds: Three from well-characterized scaffolds including heteroaryl pyrimidine (HAP), and three from novel scaffolds. Only the HAP compound induced the formation of irregular non-capsid structures (class II mode of action), while the remaining CAMs did not affect capsid gross morphology (class I mode of action). Intracellular lysates from the HepAD38 cell line, inducible replicating HBV, showed no reduction in the quantities of intracellular core protein or capsid after treatment with five compounds; however, HAP-treatment led to a reflective decrease in both. Additionally, immunofluorescence staining of compound-treated HepAD38 cells showed that all non-HAP CAMs led to a shift in the stability of HBcAg towards complete cytoplasmic staining, while HAP induced the accretion of HBcAg aggregates in the nucleus [95].

In another study, Berke et al. reported the antiviral activity of CAM BAY41-4109 and two NAs on a various panel of 54 HBV clinical isolates from genotype A-H and assessed the impact of core amino acid substitutions using site-directed mutants [96]. The amino acid substitutions were located within the CAM binding pocket and were expected to affect CAM binding based on structural modeling. Importantly, amino acid variations at these positions were rarely (<0.3%) observed as naturally occurring in public sequence databases. NAs remained fully active against these variants. They concluded that CAM BAY41-4109 generally remained fully active across genotype A-H clinical isolates. In addition, core amino acid substitutions within the CAM-binding pocket replicated in vitro and variants at specific positions were identified to reduce antiviral activity [96].

Therefore, CAMs may be a new potential therapeutic agent that affects intrahepatic core protein and possibly cccDNA, suggesting that the monitoring of HBcrAg, which reflects the amount and activity of cccDNA, will be useful to judge therapeutic effects during the HBeAg-negative phase.

### 10.2. RNA Interference (RNAi)-Based Therapeutic ARC-520

A dynamic polyconjugate (DPC) platform has been developed for the delivery of RNAi trigger molecules for therapeutic applications [97,98]. The process consists of the intravenous co-injection of cholesterol-conjugated RNAi triggers together with a membrane-active biodegradable amphipathic reversibly-masked melittin-like peptide [97,98]. The DPC platform was used in ARC-520, an RNAi-based therapeutic targeting HBV [97,99]. This included a transcript encoding HBsAg, which plays a role in changing host antiviral immune responses [97,99,100]. Because all cccDNA-derived HBV transcripts overlap at their 3′ end, the siRNAs in ARC-520 were designed to target all HBV mRNAs and reduce antigenemia, as well as enable potential host immune responsiveness and a functional cure [101].

ARC-520 in a phase II clinical study of CHB patients and a complementary study in chimpanzees chronically infected with HBV revealed a previously under-appreciated source of HBsAg; that is, HBV DNA integrated into the host genome [101]. The profile of HBcrAg was further described in 58 CHB patients treated with a combination of NA and ARC-520 targeting cccDNA-derived transcription, which was reflected by serum HBcrAg levels [39]. The initial findings of the phase II clinical trial suggested that interfering with RNA transcription reduced antigen production driven by cccDNA in HBeAg-positive patients [39].

### 10.3. Clustered Regularly Interspaced Short Palindromic Repeats (CRISPR)-CRISPR Associated Protein 9 (Cas9)

CRISPR-Cas9-mediated genome-editing technology contributes to basic genomic studies and clinical studies such as genetic correction and virus inactivation [102]. HBV is a major target for the potential application of CRISPR-Cas9 in eradicating viral DNA from human cells. However, the high stability of cccDNA makes it difficult to completely clear HBV. Highly multiplexed CRISPR-Cas9-nuclease and Cas9-nickase vector systems [103] that simultaneously target the three critical domains of the HBV genome were reported [102]. Co-transfection of an HBV-expressing plasmid and all-in-one CRISPR-Cas9 vectors [104] resulted in a significant reduction of viral replicative intermediates and extracellular HBsAg and HBeAg. In addition, successful fragmentation of the HBV genome was confirmed by DNA sequencing. Despite its high effectiveness in suppressing HBV, no obvious off-target mutations were detected by genomic cleavage detection assay and the small number of observed mutations was extremely uncommon and could only be detected by deep sequencing analysis. Thus, the all-in-one CRISPR-Cas9-nuclease and Cas9-nickase vectors present a model for the simultaneous targeting of multiple HBV domains, potentially contributing to a well-designed therapeutic approach for curing HBV patients [102]. The monitoring of HBcrAg reflecting the amount of cccDNA will be useful to judge therapeutic effects.

### 10.4. Other Anti-HBV Strategies

Recently, programmable site-specific nucleases such as zinc-finger nuclease and transcription activator-like effector nuclease [105,106] have been reported. These proof-of-concept studies showed that genome-editing technologies have the potential to eradicate HBV; however, there is a need to improve their efficiency, specificity, adaptability, and delivery. To overcome these possible off-targeting effects and to disturb the conversion to cccDNA, new methodologies using obligate heterodimeric nucleases coupled to two different targeting domains [107,108] and the targeting of non-nuclease effector domains, such as epigenetic modulators (DNA methyltransferases, histone modifiers) [109], or transcription downregulators (Krüppel-associated box) [110] are of substantial interest.

## 11. Discussion and Conclusions

In this review, we described the characteristics and clinical applications of HBcrAg, an appropriate surrogate biomarker of intrahepatic cccDNA. The amount of HBcrAg is associated with that of HBV DNA in all CHB disease states. Even in patients who achieve a “functional cure” with undetectable serum HBV DNA and HBsAg, severe complications including HBV reactivation and HCC occurrence are still reported. Because some patients achieving a “functional cure” still have detectable serum HBcrAg, prospective studies comparing the long-term outcome between HBcrAg-positive and HBcrAg-negative patients are required. The clinical practice of using HBcrAg needs further examination and discussion. Because HBcrAg showed good predictive assessment for the risk of HBV reactivation, we hope more studies will be initiated for risk stratification. Furthermore, to predict HCC occurrence more precisely using HBcrAg, we should perform careful studies using high numbers of candidate patients consisting of patients who have already developed HCC, and those who have never developed HCC. Additionally, HBcrAg might be valuable in predicting HBsAg loss under NA therapy. Although most CHB patients continue NA therapy permanently, to define the standard of NA cessation is a more attractive contribution. To choose low-risk patients for NA cessation, the role of serum HBcrAg levels should be further examined.

In the near future, it is expected that new HBcrAg assays with approximately 10 times higher sensitivity will be developed and approved. Furthermore, based on a fully-automated pretreatment technique before HBcrAg measurement, new highly sensitive HBcrAg measurements should be used to monitor HBeAg-negative patients.

Recently, the number of reports regarding HBcrAg has been increasing. To date, most reports have been published from Asian countries. For HBcrAg to be used more in clinical practice, large cohort studies should be performed in other countries in the US and Europe.

In conclusion, HBcrAg is a useful novel HBV marker. We hope that anti-HBV treatment targeting cccDNA will be put to practical use soon. Because serum HBcrAg reflects the amount and replication activity of intrahepatic cccDNA, HBcrAg will be generalized for the disease progression and treatment responses of CHB patients. To use HBcrAg for many aspects of HBV clinical practice, various trials and studies from many directions are desired.

## Figures and Tables

**Table 1:** Correlation coefficients of HBcrAg and other hepatitis B virus (HBV) markers.

No. of Patients	(a) Serum HBV DNA		(b) Intrahepatic Total HBV DNA		(c) Intrahepatic cccDNA		Year	Country Where the Patients Were Enrolled	References
	Correlation Coefficient	*p* Value	Correlation Coefficient	*p* Value	Correlation Coefficient	*p* Value			
82	Total: 0.807	<0.001					2003	Japan	[33]
	HBeAg-positive: 0.847	<0.001							
	HBeAg-negative:0.632	<0.001							
190	0.79 (HBV genotype B)	<0.001					2005	Japan	[41]
	0.87 (HBV genotype C)	<0.001							
93	0.82	<0.001	0.7	<0.001	0.664	<0.001	2007	Japan	[40]
31					0.482	<0.006	2009	Japan	[34]
138	Total: 0.69	<0.0001			0.7	<0.0001	2017	Hong Kong	[42]
	HBeAg-positive: 0.66	<0.0001							
	HBeAg-negative:0.59	<0.0001							
305			0.67	<0.001			2017	Hong Kong	[42]
79 of 82*					0.323	0.004	2019	China	[46]

**Table 2:** Relationship between HBcrAg and other HBV markers through chronic hepatitis B (CHB) natural history.

Disease Phase	HBeAg	No. of Patients	Serum HBV DNA		Serum HBsAg		Serum HBsAg-HQ		Year	Country Where the Patients Were Enrolled	References
			Correlation Coefficient	*p* Value	Correlation Coefficient	*p* Value	Correlation Coefficient	*p* Value			
Immune tolerance	Positive	30	0.45	0.013	0.47	0.0095			2015	Germany	[35]
		52	0.369	0.007	0.286	0.04	0.401	0.003	2014	Hong Kong	[25]
Immune clearance	Positive	60	0.66	<0.0001	0.53	<0.0001			2015	Germany	[35]
		105	0.484	<0.001	0.406	0.017	0.596	0.401	2014	Hong Kong	[25]
Hepatitis	Negative	50	0.74	<0.0001	0.4	0.0045			2015	Germany	[35]
		97	0.537	<0.001	0.245	<0.001	0.401	<0.001	2014	Hong Kong	[25]
Inactive/quiescent carrier	Negative	109	0.18	0.054	0.47	<0.0001			2015	Germany	[35]
		95	0.472	<0.001	0.388	<0.001	0.605	<0.001	2014	Hong Kong	[25]

**Table 3:** Clinical applications of HBcrAg.

Category	Findings	HBcrAg Level (log U/mL) and Point	References
Natural history	HBeAg seroconversion	<4.92 log U/mL during the clinical course	[25]
	HBsAg seroclearance	Undetectable (79%), 2.7 log U/mL (median of 21%) during the clinical course	[25,61]
Anti-HBV treatment	HBeAg seroconversion by PEG-IFN at 12 weeks	>8 log U/mL (no response) at the beginning of the therapy	[62]
	HBeAg seroconversion by PEG-IFN plus NA for 4 weeks followed by PEG-IFN for 20 weeks	>4.5 log U/mL (no response) at the beginning of the therapy	[68]
	No LAM resistance	<4.6 log U/mL at 6 months of treatment	[45]
	Virological relapse within 1 year of NA cessation	>3.7 log U/mL at NA cessation	[72]
	Virological relapse regardless of undetectable HBV DNA for at least 6 months	3.2–3.7 log U/mL at NA (LAM or ETV) cessation	[73,74]
	Virological relapse regardless of undetectable HBV DNA for at least 6 months	<3.4 log U/mL at LAM cessation	[74]
HCC occurrence	Incidence of HCC for treatment-naïve patients	>2.9 log U/mL during the follow-up period	[79]
	Incidence of HCC for treatment-experienced patients	>4.67 log U/mL at pre-treatment, >3.89 log U/mL at post-treatment	[82]
	HCC development during NA treatment	Detectable HBcrAg during NA treatment	[78]
	HCC recurrence within 2 years	>4.8 log U/mL at the time of HCC diagnosis	[76]
HBV reactivation	HBV reactivation by high-risk immunosuppressive therapy within 2 years	Detectable HBcrAg at baseline	[88]
HBV reinfection	High levels of post-liver transplantation cccDNA	>4 log U/mL before liver transplantation	[93]

NA: nucleos(t)ide analogues; LAM: lamivudine; ETV: entecavir; HCC: hepatocellular carcinoma.
